# JmjC histone demethylases act as chromatin oxygen sensors

**DOI:** 10.1080/23723556.2019.1608501

**Published:** 2019-05-07

**Authors:** Michael Batie, Sonia Rocha

**Affiliations:** Department of Biochemistry, Institute of Integrative Biology, University of Liverpool, Liverpool, UK

**Keywords:** Hypoxia, JmjC-histone demethylases, chromatin, HIF. transcription

## Abstract

Oxygen sensing is important in physiology but also in disease. We find that hypoxia (oxygen deficiency) triggers rapid and hypoxia-inducible factor (HIF)-independent histone methylation changes which are reversible upon reoxygenation. Hypoxia-induced histone methylation genomic distribution precedes transcriptional changes and is mimicked by specific Jumonji-C (JmjC) histone demethylase depletion. Oxygen sensing by JmjC histone demethylases is required for the cellular response to hypoxia.

Oxygenation of the earth’s atmosphere has led to the evolution of oxygen sensing and response systems. A reduction in oxygen levels (hypoxia) has implications in both physiological and pathophysiological processes, including embryogenesis, stem cell fate, and cancer^1^ In Metazoans, the hypoxia-inducible factor (HIF) pathway is central to the hypoxic response. HIFs are a family of transcription factors which coordinate many of the cellular transcriptional changes induced by hypoxia, enabling response and adaption to lower oxygen tensions^2^ HIF activation in response to hypoxia is mediated through the inhibition of 2-oxoglutarate (OG)-dependent dioxygenases (2-OGDDs), most prominently the prolyl hydroxylase domain-containing proteins (PHDs) and hypoxia-inducible factor 1 subunit alpha inhibitor (HIF1AN, also known as factor inhibiting HIF (FIH)). These enzymes require molecular oxygen as a cofactor, as well as iron and 2-OG, for activity. PHDs, and to amuch lesser extent FIH, have a low affinity (high K_m_) for oxygen, and as such display altered activity over physiologically relevant oxygen gradients^3^

There are over 60 2-OGDDs in humans, and this includes the chromatin modifying enzymes, ten eleven translocation (TET) DNA demethylases and Jumonji-C (JmjC) histone demethylases. Several studies have observed increases in histone methylation in response to hypoxia, some of which suggest this may be due to inhibition of JmjC histone demethylases^4^ Interestingly, many JmjC histone demethylases are HIF target genes and transcriptionally upregulated following longer periods of hypoxia^5^ This may in part represent a feedback mechanism to retain activity in hypoxia similar to the upregulation of egl-9 family hypoxia-inducible factor 1 (EGLN1, also known as prolyl hydroxylase domain-containing protein 2 (PHD2)) and egl-9 family hypoxia-inducible factor 3 (EGLN3, also known as prolyl hydroxylase domain-containing protein 3 (PHD3)) by HIF. Whilst the oxygen affinity for the majority of JmjC histone demethylases has not yet been established, for the ones that have, their K_m_ for oxygen is similar to that of PHD enzymes *in vitro*, demonstrating their potential to function as cellular oxygen sensors^6,7^ We,^8^ along with an accompanying study,^9^ provided evidence that chromatin is a direct sensor of oxygen though loss of JmjC histone demethylase activity.

Analysis of histone methylation levels was performed using a variety of methods in response to different lengths of hypoxia, in several cultured cell lines. This analysis revealed that hypoxia induces a rapid and robust induction of histone-3 (H3) tri-methylation (me3) on various lysine (K) residues, which preceded robust stabilization of hypoxia-inducible factor 1 subunit alpha (HIF1A, also known as HIF-1α). Similar results were seen using the hypoxia mimetics, dimethyloxaloylglycine (DMOG) and desferrioxamine (DFX), and in cells where HIF function was compromised or constitutively elevated. Furthermore, hypoxia-induced histone methylation occurred independently of reactive oxygen species, and physiological inhibitors of 2-OGDDs, namely succinate and the oncometabolite 2-hydroxyglutarate (2-HG). These data plus the rapid reversal of the response upon re-oxygenation indicate that this aspect of chromatin biology can directly sense oxygen.

To ascertain if hypoxia-induced changes to histone methylation are at genomic loci specific to the hypoxia response or more ubiquitously across the genome, we next performed genome-wide chromatin immunoprecipitation (ChIP)-sequencing on HeLa cells exposed or not to 1 hour of hypoxia and mapped the H3K4me3 and H3K36me3 landscape. These modifications are associated with open and transcriptionally active or poised loci. For both H3K4me3 and H3K36me3, the majority of predicted binding sites was shared between normoxia (normal oxygen) and hypoxia, with hypoxia upregulation of promoter H3K4me3 being enriched at hypoxia-inducible genes. Additionally, H3K4me3 was also hypoxia upregulated at enhancer sites, and some of the genes at the predicted promoter partners of these enhancers were found to be hypoxia-inducible. H3K36me3 hypoxia upregulation was enriched at HIF target genes and downregulation was enriched at hypoxia repressed genes. Following validation of acute hypoxia upregulated promoter H3K4me3 at a subset of hypoxia-inducible genes by ChIP-qPCR, we then elucidated that these changes preceded hypoxia-induced transcriptional activation at these genes. We have identified that specifically lysine demethylase 5A (KDM5A) inactivation, by siRNA depletion, mimicked hypoxia-induced gene transcription, promoter methylation, and cellular responses triggered by hypoxia.

Our results indicate that chromatin can sense oxygen at the cellular level, via JmjC histone demethylase inhibition. To further delineate the oxygen sensitivity of histone methylation marks, we analyzed histone methylation levels in response to a gradient of oxygen tensions in cells. Histone hypermethylation was induced at 10% oxygen, the same point at which HIF-1α was stabilized. This demonstrates oxygen sensing by chromatin has similar dynamics to that of the PHDs. Chakraborty *et al*^9,10^ recently reported that KDM5A, but not other H3K4me3 targeting JmjC histone demethylases (lysine demethylase 5B (KDM5B) and lysine demethylase 5C (KDM5C)) has an *in vitro* oxygen affinity comparable to that of FIH. To understand why KDM5A but not KDM5B or KDM5C may function as a cellular oxygen sensor, we performed sequence and structural analysis on these enzymes. Mutation of KDM5A on threonine (T) 30 and serine (S) 34 (JmjN domain) and methionine (M) 297 (Plant Homeodomain (PHD) 1) retained demethylase activity even at 1% oxygen. These domains do not coordinate coactivators and may increase activity via an as of yet uncharacterized mechanisms. In addition to structural differences between JmjC histone demethylases, relative levels of these enzymes in cells may help achieve specificity of oxygen-dependent responses. We thus examined available quantitative proteomic datasets and found that KDM5A is the most abundant KDM5 family member in the cell line we performed the majority of our experiments in (HeLa). Accordingly, we showed that depletion of KDM5A changed the sensitivity of cells to hypoxia. As such, reduction of KDM5A levels resulted in cells now responding to 15%, rather than 10% oxygen, thereby setting a new sensitivity threshold for oxygen in these cells.

This study provides evidence that hypoxia induces rapid changes in histone methylation, via JmjC histone demethylase inactivation, reprogramming chromatin for the subsequent gene transcriptional changes and cellular responses (Figure 1). We hypothesize that oxygen affinity coupled with levels of these enzymes will contribute to the overall sensitivity to oxygen in a given cell, promoting specificity across different tissues.

**Figure 1.**
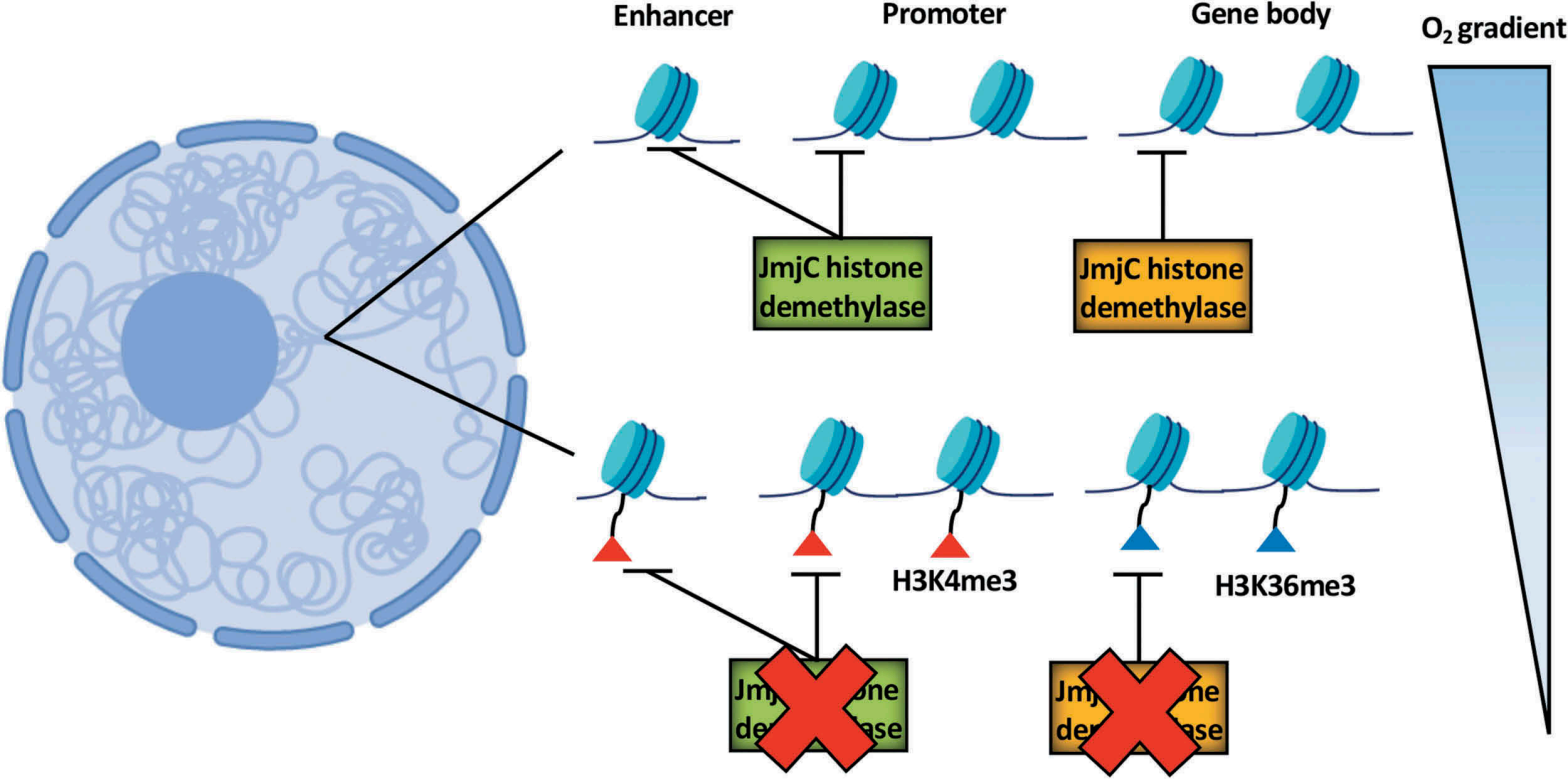
**Hypoxia-mediated redistribution of histone methylation across the genome**. Jumonji-C (JmjC), Histone-3 (H3), lysine (K), tri-methylation (me3).
